# Urinary Arsenic Species are Detectable in Urban Underserved Hispanic/Latino Populations: A Pilot Study from the Study of Latinos: Nutrition & Physical Activity Assessment Study (SOLNAS)

**DOI:** 10.3390/ijerph17072247

**Published:** 2020-03-27

**Authors:** H. Dean Hosgood, Vesna Slavkovich, Simin Hua, Madelyn Klugman, Maria Grau-Perez, Bharat Thyagarajan, Joseph Graziano, Jianwen Cai, Pamela A Shaw, Robert Kaplan, Ana Navas-Acien, Yasmin Mossavar-Rahmani

**Affiliations:** 1 Department of Epidemiology and Population Health, Albert Einstein College of Medicine, Bronx, NY 10461, USA; simin.hua@einsteinmed.org (S.H.); mklugman@mail.einstein.yu.edu (M.K.); robert.kaplan@einsteinmed.org (R.K.); yasmin.mossavar-rahmani@einstein.yu.edu (Y.M.-R.); 2 Department of Environmental Health Sciences, Mailman School of Public Health, Columbia University, New York, NY 10032, USA; vns1@cumc.columbia.edu (V.S.); mgraupe1@jhu.edu (M.G.-P.); jg24@cumc.columbia.edu (J.G.); an2737@cumc.columbia.edu (A.N.-A.); 3 Division of Molecular Pathology and Genomics, University of Minnesota Medical School, Minneapolis, MN 55455, USA; thya0003@umn.edu; 4 Collaborative Studies Coordinating Center, Department of Biostatistics, Gillings School of Global Public Health, University of North Carolina, Chapel Hill, NC 27599, USA; cai@bios.unc.edu; 5 Department of Biostatistics, Epidemiology and Informatics, University of Pennsylvania Perelman School of Medicine, Philadelphia, PA 19104, USA; ShawP@pennmedicine.upenn.edu; 6 Public Health Sciences Division, Fred Hutchinson Cancer Research Center, Seattle, WA 98109, USA

**Keywords:** arsenic, urine, Hispanic/Latino, dietary, environmental

## Abstract

Background: Hispanics/Latinos represent >15% of the United States (US) population and experience a high burden of cardiovascular disease (CVD) and diabetes. Dietary exposure, particularly to arsenic (As), may be associated with CVD and diabetes in Hispanics/Latinos. Rural populations in the US exposed to As in drinking water have increased risk of diabetes and CVD; however, little is known about the risk among urban populations with low As in water who are mostly exposed to As through food. Methods: To explore the levels of inorganic arsenic exposure (the sum of inorganic and methylated arsenic species in urine, ∑As, corrected by a residual-based method) in persons of Hispanic/Latino origin, we conducted a pilot study quantifying urinary arsenic levels among 45 participants in the Study of Latinos: Nutrition & Physical Activity Assessment Study (SOLNAS). Results: The median (interquartile range) of the urinary arsenic species (µg/L) were as follows: inorganic As 0.6 (0.4, 1.0), monomethylarsonic acid 1.2 (0.7, 1.9), dimethylarsinic acid 7.2 (4.3, 15.3), and ∑As 6.0 (4.3, 10.5). Conclusions: This study adds to the existing evidence that harmful forms of arsenic are present in this group of Hispanics/Latinos.

## 1. Introduction

Evidence from populations exposed to arsenic (As) in drinking water in rural areas has shown an increased risk of diabetes and CVD [1]. Urban populations, on the other hand, tend to have low As in drinking water and are mostly exposed to As through food. While research and regulatory efforts have addressed contaminated drinking water and occupational airborne exposures [2], the toxicological significance of As occurring in foods has received less attention.

Dietary exposure, including to As, may contribute to CVD and diabetes mortality risk in Hispanics/Latinos [3,4,5,6]. Hispanics/Latinos, who make up >15% of the United States (US) population, experience high mortality rates for cardiovascular disease (CVD) and diabetes [7,8]. Rice, grains and some juices are a major dietary source of inorganic arsenic (iAs), and fish and shellfish of organic arsenicals are common foods among Hispanics/Latinos [9,10,11]. Seafood arsenicals represent the largest quantity of dietary As in many populations [12], but are considered to be low toxicity because the majority of seafood arsenic consists of complex organic arsenical compounds. 

The sum of urinary iAs and its metabolites, monomethylarsonic acid (MMA) and dimethylarsinic acid arsenic (DMA), after removing the impact of seafood arsenicals, can be used to estimate iAs exposure in populations exposed primarily to arsenic through the diet [13]. Rice was a major source of exposure to As in the Multi-Ethnic Study of Atherosclerosis (MESA) study, and Hispanic/Latino and Asian Americans were exposed to higher iAs than Whites and Blacks [14]. Participants who ate rice ≥2 times/week had 77% higher urine arsenic compared to those that rarely/never consumed rice. However, this study used urine arsenic measurements at a single point in time, and the stability of these arsenic measures in Hispanics/Latinos is unknown. Given this background, we conducted a pilot study in SOLNAS, to explore if urinary As levels were present and potentially elevated in diverse, urban Hispanic/Latino communities. SOLNAS is an ancillary study to the parent study, the Hispanic Community Health Study/Study of Latinos (HCHS/SOL) which is a well-characterized multi-site prospective cohort study of adults aged 18–74 years of Hispanic/Latino origin [15]. The robust data and biological samples collected by this study allow for the assessment of As in those who are mainly exposed through food. We quantified urinary arsenic levels in a subsample (*n* = 45) of SOLNAS participants of HCHS/SOL participants to determine if urinary arsenic levels are detectable and elevated compared to other populations.

## 2. Materials and Methods

We conducted a pilot study examining As levels, using stored spot urine samples, in randomly selected participants (11 participants from the Bronx, Chicago and Miami and 12 from San Diego) within the SOLNAS to determine if urinary arsenic levels are detectable and elevated compared to other populations. This HCHS/SOL [15] ancillary study was comprised of 477 participants, enrolled between 2011–2012, and was designed to assess measurement error of self-reported energy, protein, sodium and potassium using the biomarker Doubly Labeled Water (DLW) and urinary biomarkers of protein, sodium and potassium [16]. This study consisted of two visits in the primary study; 20% of the participants repeated the procedures in the reliability study. Study procedures were approved by the institutional review boards of all the sites and the coordinating/reading centers. Participants were excluded for having any medical condition precluding participation, being pregnant or breastfeeding a child, weight instability, taking medication for diabetes or having extended travel plans during the study period. As part of the protocol for the DLW, spot urine was collected once pre-DLW dosing and two samples were collected post-DLW dosing. Leftover spot urine from the DLW dosing was used for this pilot study. 

Participants for this pilot study were selected at random across the site without additional predetermined criteria. SOLNAS spot urine came from a random subset of 20 participants (five from each HCHS/SOL site) who had their SOLNAS samples collected 12 days after baseline (SOLNAS Visit 1) and six months after SOLNAS Visit 1 from the reliability study. Finally, blinded duplicates from three participants were included to assess the QC measures of the laboratory. In total, urinary arsenic was quantified in 88 samples (85 after removing the three duplicates from the QC participants) from 45 participants at baseline. 

### 2.1. Laboratory Analyses

Total urine As was quantified at Columbia University by inductively coupled plasma mass spectrometry (ICPMS) using a Perkin–Elmer NexION 350S ICP-MS (PerkinElmer, Waltham, MA USA) that is highly automated. Urine As species were analyzed on a Perkin–Elmer high performance liquid chromatography (HPLC) Series 200 Pump and Series 200 Autosamplers coupled to a Perkin–Elmer ELAN DRC II ICPMS. HPLC for speciation of iAs, MMA, DMA and arsenobetaine has excellent separating power, which, coupled with the elemental specificity and high sensitivity of ICP-MS, enables As species detection without online digestion of organic forms. Importantly, to minimize the percentage of samples undetectable for iAs, we treated the urine sample with hydrogen peroxide to convert arsenite to arsenate, a previously validated method [17], and reported total iAs as the overall biomarker for inorganic arsenic. The urine sample was then diluted (1:5) with Mobile Phase (10mM Ammonium Nitrate +10mM Ammonium Phosphate, pH 9.0) and we ran it through the column (Anion Exchange, Hamilton PRP-X100) at a flow rate of 2ml/min, at room temperature. The effluent from the liquid chromatography column was directly connected to a nebulizer with PEEK tubing. Data quantification and calibration was achieved via TotalChrom 6.3 (PerkinElmer, Waltham, MA, USA), which offers powerful and flexible data review. Total As (by ICPMS) and As species (by HPLC/ICPMS) determinations were run sequentially. For this manuscript, we selected the As species to estimate exposure to As not derived from seafood. To correct for urine dilution, we measured urine creatinine using the Jaffe reaction method and specific gravity using Total Solids Refractometer model Reichtert TS 400 (Reichert Analytical Instruments, Depew, NY, USA). The comparisons by participant characteristics were similar for data in µg/L and µg/g creatinine, so data in µg/L are shown. For our highly sensitive methods, the limit of detection (LOD) of each arsenic species is 0.1 µg/L. Samples below the LOD were replaced by the LOD divided by the square root of two.

### 2.2. Statistical Analyses 

We calculated the arsenic exposure not derived from seafood (∑As) for each participant, using a validated method [13]. Briefly, ∑As is the sum of inorganic and methylated arsenic species estimated by applying a residual-based method to remove the impact of seafood arsenicals using urine arsenobetaine, a seafood arsenical that correlates with other seafood arsenicals. The beta coefficients (and standard errors) for the calibration models were as follows: iAs: β_0_ = −0.59 (SE = 0.11), β_1_ = 0.14 (SE = 0.07); DMA: β_0_ = 1.81 (SE = 0.09), β_1_ = 0.28 (SE = 0.05); MMA: β_0_ = −0.01 (SE = 0.09), β_1_ = 0.13 (SE = 0.05). For reference, the unadjusted urinary As levels (inorganic and methylated) by subgroups are provided in Supplementary Table S1. To assess the distribution of arsenic exposure by participant characteristics, we computed the medians (and interquartile ranges (IQR)) of urinary concentrations of total arsenic, for the sum of inorganic and methylated arsenic species. Participant characteristics included the study site, participants’ Hispanic/Latino background (Caribbean includes Cuban, Dominican, Puerto Rican; Mainland includes Mexican, Central American and South American), rice intake (above versus below median intake, 79 g per day, assessed using a 24 h dietary recall) and obesity status (BMI ≥ 30 kg/m^2^ are obese). For this analysis, we utilized samples from the baseline visit (*n* = 45). Stratified analyses were also conducted using existing biomarker data [folate, measured in serum using the Siemens ADVIA Centaur Folate assay (Siemens Healthcare Diagnostics, Deerfield IL), stratified by median level (15.8 ug/L)]. To assess stability of urinary arsenic levels across the three visits (baseline, follow-up at 6–12 days, and follow-up at 6–12 months), we used mixed effects models to estimate intra-class correlations and 95% confidence intervals (CI). Urine arsenic concentrations for total arsenic and the arsenic species were right-skewed and log-transformed for the analyses. We compared the difference in distributions of arsenic levels by subgroups using the Wilcoxon rank sum test for two groups and the Kruskal–Wallis test for more than two groups. Statistical analyses were performed using SAS software (version 9.4) (SAS, Cary, NC, USA) and R (version 3.2.4) (R Core Team). All tests were two-sided with *p* < 0.05 considered to be statistically significant. 

## 3. Results

Subjects were a median age of 45 years (IQR 36, 51) and were evenly distributed by sex and study site (Table 1). The most predominant Hispanic/Latino background was Mexican, comprising 31% of the sample, followed by Puerto Rican (27%), and Cuban (22%). The majority of the subjects were born outside of the 50 US States (73%). The median BMI was 28 kg/m^2^ (IQR 25, 30).

Other than undetectable iAs in one sample from SOLNAS Visit 1 and two samples from SOLNAS Visit 2, all samples had detectable measurements of each of the urinary As species. The mean ∑As level of urine samples from Visit 1 was 9.58 µg/L (range 1.99–69.30) corresponding to 6.80 µg/g creatinine (range 1.59–25.11) (Table 2). Changes in levels of urinary As species over up to 12 months in the twenty subjects with repeated measurements were generally modest (Supplementary Table S2). The intra-class correlation (ICC) for ∑As across all three visits was 0.48 (95% CI = 0.27–0.70) (Table 3). Between the first and second visit (6–12 days later), the ICC was higher (ICC = 0.62; 95% CI = 0.37–0.82) compared to the ICC between the first and third visit (6–12 months later) (ICC = 0.42 95% CI = 0.15–0.76). Levels were fairly consistent between the baseline visit and the 6–12 month follow-up visit for %DMA (ICC = 0.71; 95% CI = 0.49–0.86), %MMA (ICC = 0.72; 95% CI = 0.50–0.87), and % iAs (ICC = 0.65; 95% CI = 0.41–0.84). 

**Figure 1:**
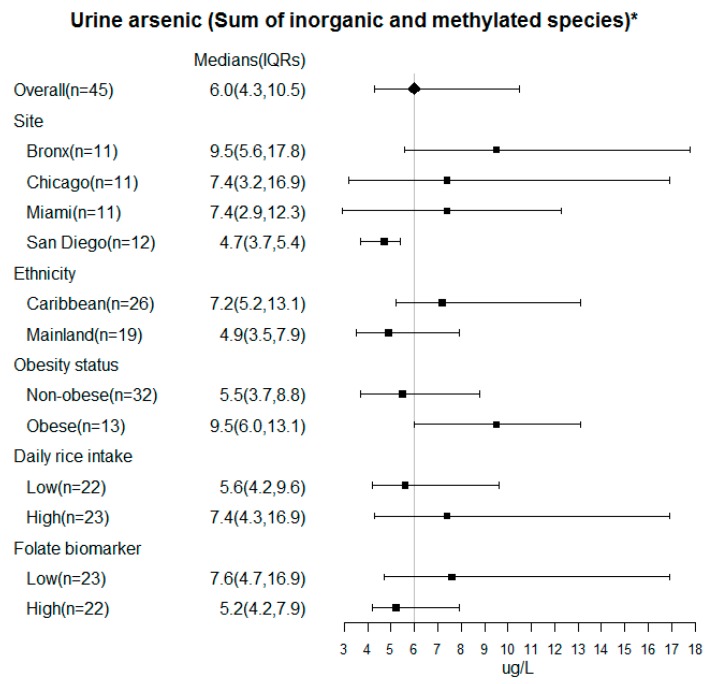
Urinary arsenic not derived from seafood (∑As) in analytes SOLNAS ancillary to HCHS/SOL by site, obesity, rice intake and folate biomarker. * Estimated sum of inorganic and methylated arsenic species applying a residual-based method to remove the impact of seafood arsenicals using urine arsenobetaine, a seafood arsenical that correlates with other seafood arsenicals, based on a method previously validated in Jones et al. (2016). Caribbean includes Cuban, Dominican, Puerto Rican and Mainland includes Mexican, Central American and South American. BMI ≥ 30 kg/m^2^ are obese. The rice intake and folate biomarker dichotomized at median 79 g per day and 15.8 ug/L.

Further, in the analysis of the samples from the baseline visit (*n* = 45), the overall median (IQR) ∑As level was 6.0 (4.3, 10.5) µg/L in SOLNAS (Figure 1), with higher levels observed in The Bronx (9.5 (5.6, 17.8) µg/L) compared to other sites (Chicago: 7.4 (3.2, 16.9) µg/L; Miami 7.4 (2.9, 12.3) µg/L, San Diego 4.7 (3.7, 5.4) µg/L) (*p* = 0.05). Although not significant, there was a suggestion that low levels of blood folate (*p* = 0.10), compared to high levels, were associated with higher urine arsenic levels. Participants with high daily rice intake (*p* = 0.52) and who were obese (*p* = 0.17) had increased urine arsenic levels compared to those with low daily rice intake and those who were not obese, respectively. 

## 4. Discussion

This pilot study, using a highly sensitive analytical method, detected urinary iAs species in all but one of the baseline samples of the SOLNAS ancillary study to HCHS/SOL. These findings indicate that urban Hispanics/Latinos are exposed to substantial iAs and its metabolites, despite the theoretical lack of drinking water exposure. In addition, levels remained relatively stable between the baseline visit and the 6–12 month follow-up visit, supporting that the relative proportion of the species in the urine is an adequate biomarker of As intake and metabolism in HCHS/SOL. Our findings are consistent with urinary arsenic measurements from MESA, a population-based sample of 6814 men and women aged 45–84 from six US field centers (Winston-Salem, North Carolina; New York, New York; Baltimore, Maryland; St. Paul, Minnesota; Chicago, Illinois; and Los Angeles, California) [14]. The IQRs of our observed ∑As level in samples from Visit 1 (4.3, 10.5 µg/L) overlap with the IQRs of ∑As levels in participants in MESA (1.7, 5.5 µg/L), although the median levels in this study were higher, potentially reflecting the higher rice intake in the SOLNAS population compared to MESA, which included White, Black, Hispanic/Latino, and Chinese subjects. In support of this hypothesis, higher urinary iAs levels in MESA participants of Hispanic/Latino and Asian ethnicity were observed compared to White and Black MESA participants [14]. Indeed, when comparing urinary As species levels of the present study versus those of Hispanic/Latino subjects in MESA (*n* = 75), the IQRs usually overlapped, although the median levels of each arsenic species were higher in our study (Supplementary Table S3). Of note, Hispanics/Latinos from New York in the MESA study had higher arsenic exposure compared to Hispanics/Latinos from other cities in MESA [14]. Our study also observed the suggestion of higher levels of urinary As among New York participants. These findings in MESA and SOLNAS indicate that regional variations in As could be due to variations in ethnic backgrounds and cultures leading to different dietary habits across the US. Interestingly, there was less variability in ∑As levels in San Diego participants compared to participants from other sites, which may be due to statistical chance or less variation in diet in those participants. Future studies should attempt to replicate these findings in large samples to determine a more precise estimate of urine arsenic species in urban Hispanic/Latino populations. 

Obese participants had higher ∑As levels compared to non-obese participants. Both arsenic exposure and arsenic methylation are associated with oxidative stress and the generation of reactive oxygen species (ROS), which is a risk factor for obesity and, in turn, a risk factor for diabetes and cardiovascular disease. Our data also suggest that high versus low folate, measured by biomarker and dietary data, may be associated with arsenic levels. Randomized clinical trials have demonstrated that folic acid supplementation facilitates As methylation, increasing urinary As excretion and lowering blood As levels [18,19]. This study adds evidence of an association between diets rich in folate and lower levels of ∑As, which likely occurs because of increased metabolism and excretion. 

Our use of biomarkers and robust dietary recall to quantify dietary exposures to As and folate, is a major strength of our study. Another strength is our use of a residual-based method to estimate As exposure and metabolism by food source, as it is a sensitive method to separate toxic versus less toxic forms of As exposure. Our study was limited, however, by our sample size of 45 participants being underpowered to detect important potential factors associated with As. For example, the relationship between rice intake and obesity is likely multifactorial and our study cannot tease apart mediators of this relationship due to As or other causes (i.e., calorie intake). Future research in HCHS/SOL should assess if low-dose, chronic As exposure is prospectively associated with obesity, cardiovascular disease and diabetes.

## 5. Conclusions

This study demonstrates relatively elevated levels of urinary iAs a known toxicant and carcinogen in urban Hispanic/Latino populations. This small study is part of a prospective cohort that, in the future, could assess if low to moderate arsenic exposures through food sources are associated with cardiometabolic diseases in an understudied population. 

## Figures and Tables

**Table 1:** Baseline characteristics of the selected pilot sample from the Study of Latinos: Nutrition & Physical Activity Assessment Study (SOLNAS) ancillary study to the Hispanic Community Health Study/Study of Latinos (HCHS/SOL).

Characteristic	Women	Men	All
	*n* = 22	*n* = 23	*n* = 45
Age (years), Median (IQR)	45.0 (36.0, 57.5)	45.0 (37.5, 50.0)	45.0 (36.0, 51.0)
BMI (kg/m2), Median (IQR)	28.6 (25.8, 29.8)	27.4 (24.8, 30.8)	28.3 (25.0, 30.4)
Center, *n* (%)			
Bronx	4 (18.2)	7 (30.4)	11 (24.4)
Chicago	6 (27.3)	5 (21.7)	11 (24.4)
Miami	4 (18.2)	7 (30.4)	11 (24.4)
San Diego	8 (36.4)	4 (17.4)	12 (26.7)
**Hispanic/Latino Background, *n* (%)**			
Central American	2 (9.1)	1 (4.3)	3 (6.7)
Cuban	4 (18.2)	6 (26.1)	10 (22.2)
Dominican	2 (9.1)	2 (8.7)	4 (8.9)
Mexican	8 (36.4)	6 (26.1)	14 (31.1)
Puerto Rican	5 (22.7)	7 (30.4)	12 (26.7)
South American	1 (4.5)	1 (4.3)	2 (4.4)
Nativity, *n* (%)			
Not born in 50 US States	17 (77.3)	16 (69.6)	33 (73.3)
Born in 50 US States	5 (22.7)	7 (30.4)	12 (26.7)

**Table 2:** Mean, median, range, and number below limit of detection (LOD) of urinary arsenic (As) species for each analyte by visit in SOLNAS ancillary to HCHS/SOL ^α^.

Arsenic	SOLNAS Visit	Mean (SD)	Median (IQR)	Minimum	Max	# Below LOD
u∑As †, µg/L	1	28.46 (47.42)	16.20 (7.20, 24.40)	2.20	227.20	0
	2	11.71 (8.00)	11.90 (4.38, 17.28)	1.30	26.20	0
	3	20.50 (18.71)	12.65 (10.00, 26.32)	2.10	78.90	0
∑As ¥, µg/L	1	9.58 (10.75)	6.04 (4.30, 10.46)	1.99	69.30	N/A
	2	7.34 (5.94)	5.43 (2.77, 12.77)	1.03	19.18	N/A
	3	9.80 (5.94)	8.54 (5.75, 11.99)	2.70	24.84	N/A
Inorganic As (iAs), µg/L	1	0.91 (0.83)	0.60 (0.40, 0.97)	<LOD	3.37	1
	2	0.71 (0.64)	0.58 (0.18, 0.87)	<LOD	2.19	2
	3	1.11 (0.94)	0.74 (0.58, 1.31)	0.19	4.11	0
Monomethylated As (MMA), µg/L	1	2.08 (5.32)	1.16 (0.69, 1.86)	0.19	36.41	0
	2	1.09 (0.98)	0.86 (0.44, 1.45)	0.13	4.11	0
	3	1.75 (1.21)	1.38 (0.92, 2.13)	0.39	4.83	0
Dimethylated As (DMA), µg/L	1	13.26 (22.01)	7.18 (4.29, 15.27)	1.25	148.86	0
	2	7.05 (5.17)	5.69 (3.13, 11.32)	0.78	18.95	0
	3	10.26 (6.24)	9.02 (6.36, 10.71)	1.73	26.76	0
iAs %	1	8.78 (3.73)	8.28 (6.51, 11.03)	0.22	20.09	N/A
	2	8.67 (4.07)	8.98 (6.28, 10.00)	0.50	20.86	N/A
	3	9.58 (3.84)	8.26 (7.37, 11.24)	5.36	21.30	N/A
MMA%	1	12.26 (4.96)	12.25 (9.12, 14.69)	2.49	26.32	N/A
	2	12.69 (4.28)	11.59 (9.75, 13.71)	7.47	21.85	N/A
	3	13.60 (5.22)	13.21 (10.51, 17.23)	5.84	23.12	N/A
DMA%	1	80.31 (7.66)	79.56 (76.22, 83.93)	59.81	96.09	N/A
	2	79.69 (5.95)	80.13 (77.34, 83.21)	68.20	89.79	N/A
	3	78.12 (7.89)	78.59 (73.14, 83.75)	64.36	89.78	N/A

^α^*n* = 45 for Visit 1, *n* = 20 for Visits 2 and 3. SOLNAS visits correspond to the first visit for the SOLNAS ancillary study, second visit (6–12 days later) and third visit (6–12 months later). **^†^** Sum of iAs, monomethylarsonic acid (MMA) and dimethylarsinic acid (DMA) before correcting for seafood As; ^¥^ arsenic not derived from seafood estimated using sum of inorganic and methylated arsenic species applying a residual-based method to remove the impact of seafood arsenicals using urine arsenobetaine, a seafood arsenical that correlates with other seafood arsenicals (previously validated in Jones et al. (2016)).

**Table 3:** Intra-class correlations and 95% confidence intervals of repeated measurements of urinary arsenic (As) species based on the time from the first visit in the SOLNAS ancillary study to HCHS/SOL.

As Species	All Visits ^†^ (*n* = 85)	Second vs. First (*n* = 65)	Third vs. First (*n* = 65)
Log-∑As *	0.48 (0.27–0.70)	0.62 (0.37–0.82)	0.42 (0.15–0.76)
Inorganic As (iAs) %	0.36 (0.15–0.63)	0.20 (0.02–0.75)	0.65 (0.41–0.84)
Monomethylarsonic acid (MMA) %	0.74 (0.57–0.85)	0.71 (0.48–0.86)	0.72 (0.50–0.87)
Dimethylarsinic acid (DMA) %	0.67 (0.49–0.81)	0.57 (0.31–0.8)	0.71 (0.49–0.86)

Except for iAs%, MMA% and DMA%, other metals were log-transformed; ^†^ three visits total, the second 6–12 days after and the third 6–12 months after the first; * total arsenic not derived from seafood (∑As) estimated using sum of inorganic and methylated arsenic species applying a residual-based method to remove the impact of seafood arsenicals using urine arsenobetaine, a seafood arsenical that correlates with other seafood arsenicals (previously validated in Jones et al. (2016)).

## Supplementary Materials

The following are available online at https://www.mdpi.com/1660-4601/17/7/2247/s1, Table S1. Unadjusted Urine Arsenic levels (inorganic and methylated) by subgroups. Table S2. Mean, median, range of absolute change in urinary arsenic (As) species for each analyte by visitα among 20 subjects with repeated measurements in select cohort of Hispanic Community Health Study/Study of Latinos (HCHS/SOL). Table S3. Comparison of median (IQR) of urinary arsenic (As) species for each analyte in select cohort of Hispanic Community Health Study/Study of Latinos (HCHS/SOL) and Hispanic/Latino participants in the MESA study ^1^.
