# Supplementary material for: Urinary Arsenic Species are Detectable in Urban Underserved Hispanic/Latino Populations: A Pilot Study from the Study of Latinos: Nutrition & Physical Activity Assessment Study (SOLNAS)
Source: Int J Environ Res Public Health. 2020 Mar 27;17(7):2247. doi: 10.3390/ijerph17072247 (PMC7178047; doi:10.3390/ijerph17072247)
Supplement: Supplementary file 1 [file ijerph-17-02247-s001.pdf]

Table S1. Unadjusted Urine Arsenic levels (inorganic and methylated) by subgroups in SOLNAS [α].

	Median(IQRs)
Overall(n=45)	9.1(5.5,18.5)
Site	
Bronx(n=11)	16.3(10.2,19.8)
Chicago(n=11)	8.1(5.9,17.5)
Miami(n=11)	10.0(6.0,20.0)
San Diego(n=12)	7.2(3.0,10.4)
Ethnicity	
Caribbean(n=26)	12.8(6.4,19.5)
Mainland(n=19)	8.7(4.0,16.3)
Obesity status	
Non-obese(n=32)	8.2(5.5,17.3)
Obese(n=13)	16.3(8.9,18.7)
Daily rice intake	
Low(n=22)	9.3(4.7,16.8)
High(n=23)	9.1(6.6,19.9)
Folate biomarker	
Low(n=23)	16.6(7.1,19.9)
High(n=22)	7.5(5.0,13.8)

[α] Arsenic not derived from seafood estimated using sum of inorganic and methylated arsenic species applying a residual-based method to remove the impact of seafood arsenicals using urine arsenobetaine, a seafood arsenical that correlates with other seafood arsenicals (previously validated in Jones et al. Am J Epidemiol 2016).

Table 2. Mean, median, range of absolute change in urinary arsenic (As) species for each analyte by visit [α] among 20 subjects with repeated measurements in select cohort of Hispanic Community Health Study/Study of Latinos (HCHS/SOL).

	SOLNAS Visit	Mean (SD)	Median (IQR)	Minimum	Max
**∑As*, μg/L**	2 vs 1	-1.97 (4.97)	-1.92 (-3.46, -0.58)	-12.21	11.66
	3 vs 1	0.49 (6.88)	-0.79 (-4.84, 5.16)	-10.74	15.42
**Inorganic As (iAs), μg/L**	2 vs 1	-0.18 (0.73)	-0.10 (-0.30, -0.04)	-2.45	1.32
	3 vs 1	0.22 (1.01)	0.05 (-0.28, 0.50)	-2.21	3.05
**Monomethylated As (MMA), μg/L**	2 vs 1	-0.46 (1.22)	-0.28 (-0.91, -0.12)	-4.17	2.94
	3 vs 1	0.20 (1.53)	-0.02 (-0.68, 0.97)	-3.47	3.23
**Dimethylated As (DMA), μg/L**	2 vs 1	-4.73 (8.32)	-3.75 (-5.73, 0.03)	-27.50	9.83
	3 vs 1	-1.52 (9.97)	-1.64 (-6.53, 3.90)	-20.93	15.81
**iAs %**	2 vs 1	0.03 (5.03)	0.33 (-2.66, 1.85)	-8.61	14.76
	3 vs 1	0.95 (3.04)	0.93 (-0.81, 2.33)	-5.33	9.07
**MMA%**	2 vs 1	-0.02 (3.69)	-0.12 (-2.49, 1.71)	-6.73	7.86
	3 vs 1	0.90 (3.74)	0.44 (-1.03, 4.25)	-6.95	7.98
**DMA%**	2 vs 1	-0.35 (6.73)	0.18 (-4.72, 3.51)	-13.76	12.32
	3 vs 1	-1.91 (5.66)	-1.32 (-5.48, 1.57)	-12.92	8.71

[α] SOLNAS visits correspond to 1st visit for SOLNAS Ancillary Study, second visit (6-12 days later) and third visit (6-12 months later). *Arsenic not derived from seafood (∑As) estimated using sum of inorganic and methylated arsenic species applying a residual-based method to remove the impact of seafood arsenicals using urine arsenobetaine, a seafood arsenical that correlates with other seafood arsenicals (previously validated in Jones et al. Am J Epidemiol 2016).

Table 3. Comparison of median (IQR) of urinary arsenic (As) species for each analyte in select cohort of Hispanic Community Health Study/Study of Latinos (HCHS/SOL) and Hispanic/Latino participants in the MESA study[1].

Urine arsenic measures, μg/L	HCHS/SOL (n=45) Median (IQR)	MESA Hispanic/Latinos (n=75) Median (IQR)
**∑As**	6.0 (4.3, 10.5)	3.9 (2.4, 6.2)
**Inorganic As (iAs)**	0.6 (0.4, 1.0)	0.1 (0.1, 0.4)
**Monomethylarsonic acid (MMA)**	1.2 (0.7, 1.9)	0.4 (0.2, 0.6)
**Dimethylarsinic acid (DMA)**	7.2 (4.3, 15.3)	3.1 (2.1, 5.3)

ΣAs: sum of inorganic and methylated arsenic species. [1] Jones, M.R., et al., Ethnic, geographic and dietary differences in arsenic exposure in the multi-ethnic study of atherosclerosis (MESA). J Expo Sci Environ Epidemiol, 2018.
